# Correlation of Plasma Catestatin Level and the Prognosis of Patients with Acute Myocardial Infarction

**DOI:** 10.1371/journal.pone.0122993

**Published:** 2015-04-07

**Authors:** Dan Zhu, Hong Xie, Xinyu Wang, Ying Liang, Haiyi Yu, Wei Gao

**Affiliations:** Department of Cardiology, Peking University Third Hospital, Key Laboratory of Cardiovascular Molecular Biology and Regulatory peptides, Ministry of Health (Peking University Third Hospital), Beijing, China; University of Florida, UNITED STATES

## Abstract

Catestatin is a peptide which is a potent inhibitor of catecholamine secretion and played essential functions in the cardiovascular system. Previous research found that dramatic changes of catestatin were associated with hemodynamics in acute myocardial infarction (AMI) during the first week after the AMI symptoms onset, but whether catestatin is also involved in the pathophysiological progression after AMI and then a predictor for outcomes is not clear. The aim of this study is to determine the correlation of plasma catestatin levels at different time points and the prognosis of AMI. 100 participants recruited were all patients with AMI, all of who received successful primary percutaneous coronary intervention (PCI) within 12h from the AMI symptom onset in our center; the concentrations of plasma catestatin were evaluated from blood samples of those 100 participants. Subsequent 65 months' follow-up was performed after discharging to evaluate cardiac adverse events and the association between catestatin levels and prognosis of AMI was examined. We confirmed the dramatic change of catestatin concentrations in the first week of AMI, and the levels of catestatin on D3 were much higher in adverse events group than those in non-adverse events group (p<0.0001), but the ratio of D7/D3 was significantly lower. In addition, the Kaplan-Meier analysis showed that the groups in which the levels on D3 were higher (p<0.0001) and the ratios of D7/D3 were lower (p<0.0001), patients trended to be more susceptive to adverse events after AMI. Furthermore, according to the analysis, we surmised catestatin level on D3 as an appropriate predictor for outcomes in patients with AMI with good specificity as well as sensitivity. All of the evidence confirmed that catestatin plays an important role in the progress of AMI, and may act as a promising target for prognostic prediction.

## Introduction

Myocardial infarction is a major cause of morbidity and mortality worldwide. More than 3 million people each year are estimated to have an acute ST-elevation myocardial infarction (STEMI), with more than 4 million having a non-ST-elevation myocardial infarction (NSTEMI) [1]. Although deaths due to acute AMI have decreased rapidly in these days because of timely revascularization and multiple drug treatments, the adverse events such as heart failure and malignant arrhythmia that occur after AMI are still major causes of the poor long-term prognosis of patients with AMI [2,3]. Therefore, evaluation of adverse events is essential in predicting the prognosis of patients with AMI and methods to evaluate these processes are of great clinical importance.

AMI is associated with a complex pattern of neurohumoral activation in which catecholamines play an important role in both onset and course of AMI [4,5]. Catestatin is a 21-amino acid residue, cationic and hydrophobic peptide which is generated endogenously by proteolytic cleavage of its precursor chromogranin A (CHGA) [6], a protein found in secretory granules of chromaffin cells, postganglionic sympathetic neurons [7] and heart cells itself [8], where it was co-stored and co-released with catecholamines. Catestatin can inhibit catecholamine release from chromaffin cells and noradrenergic neurons [9,10] and induce desensitization of catecholamine release induced by nicotine [11]. In recent years, catestatin has been found to exert effects on cardiovascular system, for example, it displayed potent vasodilatory effect in rate which was mediated, at least in part, by augmented release of histamine [12], the possible mechanism of which involves inhibitory G proteins [13]. Another research in humans also proved that increasing concentrations of catestatin resulted in dose dependent vasodilation [14]. Meng et al. recently demonstrated that the plasma catestatin levels after the onset of AMI might be associated with left ventricular remolding (LVR) [15], those with LVR had a significantly higher level of plasma catestatin on admission, on day 3 and day 7. Furthermore, our study before proved its predictive effect to detect stage B failure [16]. Taken together, these data point to catestatin as a novel regulator of blood pressure and cardiac function.

On the basis of these cardiovascular effects of catestatin, we keep to ask whether catestatin exerts cardioprotective influence under Ischemia/Reperfusion (I/R) condition. Cardioprotection includes endothelial and adrenergic components [17], and these were proved to be affected by catestatin via anti-endothelian-1/pro-nitric oxide and anti-adrenergic actions, respectively [18,19]. Following this hypothesis, researchers used Langendorff reperfused rat heart and isolated cardiomyocytes and demonstrated the cardioprotective role for catestatin, which appears mainly due to a direct reduction of post-ischemic myocardial damages and dysfunction, rather than to an involvement of adrenergic terminals and/or endothelium [20], other researches also found that catestatin reduced myocardial I/R injury involving PI3K/AKT, PKCs, mitochodrial KATP channels and ROS signaling [21], and it can also increase the expression of anti-apoptotic and pro-angiogenetic factors in the post-ischemic hypertrophied heart of spontaneously hypertensive rats [22]. Due to the multiple and important functions of catestatin in the cardiac system, it provides a rationale for the hypothesis that catestatin might be a good marker or predictive factor in the pathophysiological process of AMI, especially for the outcomes. Since the role of catestatin in this aspect is yet to be addressed, in this study, we aimed to evaluate the plasma catestatin levels in patients with AMI within the first week, and determine the association between plasma catestatin levels and the adverse outcomes after AMI in our 65 months' follow-up study.

## Materials and Methods

### Study population

This study was approved by the ethics review boards of Peking University Health Science Center. Written informed consent was obtained from the study population. A total 117 consecutive patients with the first AMI admitted to the Department of Cardiology, Peking University Third Hospital, from June 2008 to April 2009 was included, and 17 of them were lost to follow-up, so we just analyzed the other 100 patients in the rest of the study. All patients received successful PCI within 12h from the AMI symptom onset. The STEMI was diagnosed according to the American College of Cardiology/American Heart Association guideline in 2004 [23]. The study excluded patients with chronic obstructive pulmonary disease, significant kidney or hepatic diseases, tumor, and infectious disease. During the same study period, all 30 subjects who were admitted to the same hospital because of atypical chest pain but with normal coronary arteries confirmed by coronary angiography were included as controls. Resting blood pressure and heart rates (HRs) were measured at the same time when blood samplings were performed in triplicate in the supine position, using an oscillometric device (Philips IntelliVue MP20, Germany).

### Blood sampling

At the acute phase of AMI, blood samples were obtained from an antecubital vein without stasis in all patients immediately after admission to the emergency room (ER), and in the morning of the third (D3) and seventh day (D7) after AMI (n = 100). In the control subjects, blood samples were obtained from the antecubital vein in the morning of the same day when angiography was performed (n = 30). The blood samples, anticoagulated with ethylenediaminetetraacetic acid (EDTA), were immediately centrifuged at 3000rpm for 10min at 4°C. An aliquot of the EDTA plasma was stored at −80°C till analysis. Repeated freeze-thaw cycles were avoided.

### Assays

Plasma levels of catestatin were measured by enzyme-linked immunosorbent assay (ELISA) according to the manufacturer’s instruction (Cat. # EK-053-29, ELISA kit, Phoenix Pharmaceutical Inc., Burlingame, CA). The minimal detection limits for catestatin was 0.06ng/ml. These assays were performed by an investigator blinded to the sources of the samples.

### Follow-up measurements and end points

The cardiac adverse events were measured at follow-up, within 65 months after study participation. 17 patients were lost to follow-up. The end points including death from cardiovascular causes, readmission with ACS or admission with CHF were evaluated within 65 months (54.98±19.20 months). Information on end points was collected from telephone interviews with patients or relatives of patients, hospital databases and patient case notes. The definition of readmission with ACS is: (1) STEMI, ST elevation>1 mm in two limb leads or>2 mm in leads V1–V6 or new left bundle branch block; (2) NSTEMI, no ST elevation on ECG despite elevated troponin T>0.01μg/ml; and (3) unstable angina, ischemic chest pain lasting more than 30 min with no evidence of myocyte necrosis or ST elevation. CHF was defined as hospitalization for a clinical syndrome involving at least two of the following: paroxysmal nocturnal dyspnea, orthopnea, elevated jugular venous pressure, pulmonary crackles, third heart sound, and cardiomegaly or pulmonary edema on chest X-ray. These clinical signs and symptoms must have represented a clear change from the normal clinical status, requiring intravenous diuretics, inotropic support or vasodilator therapy.

### Statistics

All results are expressed as the mean ± SD. Student's unpaired t-tests were used to compare the catestatin levels between control subjects and patients with AMI on ER, D3 and D7, as well as the differences between adverse events group and non-adverse events group. A chi-square test was used to determine differences between groups. On multivariable Cox proportional hazard analyses, the main models were adjusted for age, gender, and variables considered might reflect the severity of AMI at baseline. Multivariable Cox proportional hazard were performed as stepwise regressions with backward elimination. Kaplan-Meier analysis was performed on the non-adverse events occurrence rates stratified into 2 groups based on the median value, and the differences between the curves were analyzed by long-rank test. The sensitivity and specificity of D3, D7 and D7/D3 for predicting adverse events were determined, and receiver operating characteristics (ROC) curves were constructed. Spearman or Pearson correlation was used to identify the bivariate correlations. A value of p<0.05 was considered significant.

## Results

### Baseline clinical characteristics

The clinical characteristics and laboratory findings of patients with AMI and control subjects are summarized in Table 1. Totally, 100 patients with AMI (age, 62.1±14.0years; sex, 84.0% male) and 30 control subjects (age, 60.0±10.4years; sex, 76.6% male) were included. Risk factor profiles and most baseline clinical parameters were compared between the two groups: the heart rate, Hs-CRP and fasting glucose of AMI patients were significantly higher than those of control subjects, but other parameters stayed the same such as age, gender, blood pressure etc.

**Table 1:** Patient characteristics and laboratory findings at baseline.

	AMI	Controls	
Variables	n = 100	n = 30	p value
**Age (years)**	62.1±14.0	60.0±10.4	0.41
**Male (%)**	84.0	76.6	0.41
**Body mass index (kg/m2)**	25.5±3.4	27.0±5.7	0.16
**Systolic blood pressure (mmHg)**	134.3±17.9	130±13.5	0.21
**Diastolic blood pressure (mmHg)**	77.1±13.1	75.0±7.4	0.41
**Heart rates (beats/min)**	82.3±11.8	70.4±8.7	<0.0001
**Diabetes mellitus (%)**	22.0	23.3	1.00
**Hypertension (%)**	62.0	70.0	0.52
**Hypercholesterolemia (%)**	56.0	50.0	0.68
**Current smoking (%)**	56.0	36.7	0.09
**Beta-blocker treatment before (%)**	6.0	10.0	0.43
**Hs-CRP (mg/l)**	19.94±24.83	1.68±2.05	<0.0001
**Creatinine (μmol/l)**	97.0±17.5	101.0±23.3	0.26
**Fasting glucose (mmol/l)**	6.5±2.3	5.0±1.7	0.002
**Total cholesterol (mmol/l)**	4.83±1.18	4.73±1.08	0.70
**LDL cholesterol (mmol/l)**	3.12±0.87	3.05±1.03	0.74

Values represent mean ± SD or the percent of the patients and control subjects.

Hs-CRP, high-sensitivity C-reactive protein; LDL, low-density lipoprotein.

### Time course of the plasma catestatin levels

**Fig 1:**
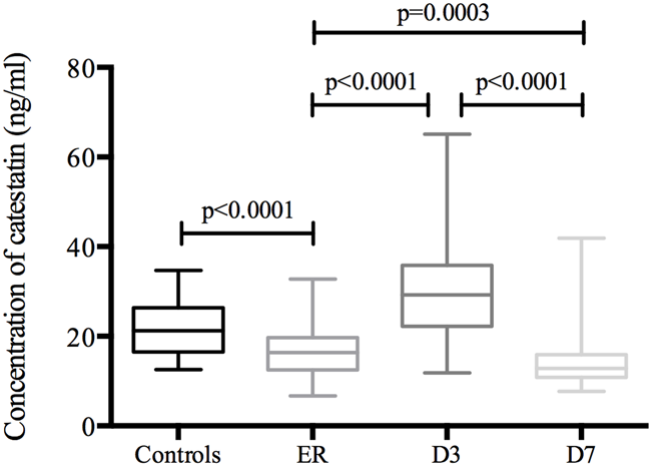
Plasma catestatin levels in controls and patients during the first week after AMI. Box plots showing the distribution of concentrations of plasma catestatin at different time points (n = 100). Boxes specify inter-quartile ranges; the horizontal bars inside the boxes indicate medians; the upper and lower whiskers get to the most distant value within the 95% of the distribution. The p-values for the comparison of the groups are also indicated. Control (21.8±6.3ng/ml); ER: emergency room (16.7±5.4ng/ml); D3: the third day after AMI (30.9±12.1ng/ml); D7: the seventh day after AMI (13.9±5.2ng/ml).

Compared with the control group (21.8±6.3ng/ml, n = 30), plasma catestatin concentrations were significantly decreased on ER (p<0.0001). Among the three time points (ER, D3 and D7), the highest concentrations of catestatin in patients with AMI were found on D3 (30.9±12.1ng/ml), which were higher than those obtained on ER (16.7±5.4ng/ml, p<0.0001), whereas the concentrations remained low at 1 week (13.9±5.2ng/ml, p<0.0001), which were also lower than the values on ER (p = 0.0003) (Fig 1).

### Patient Characteristics according to the occurrence of adverse events

**Fig 2:**
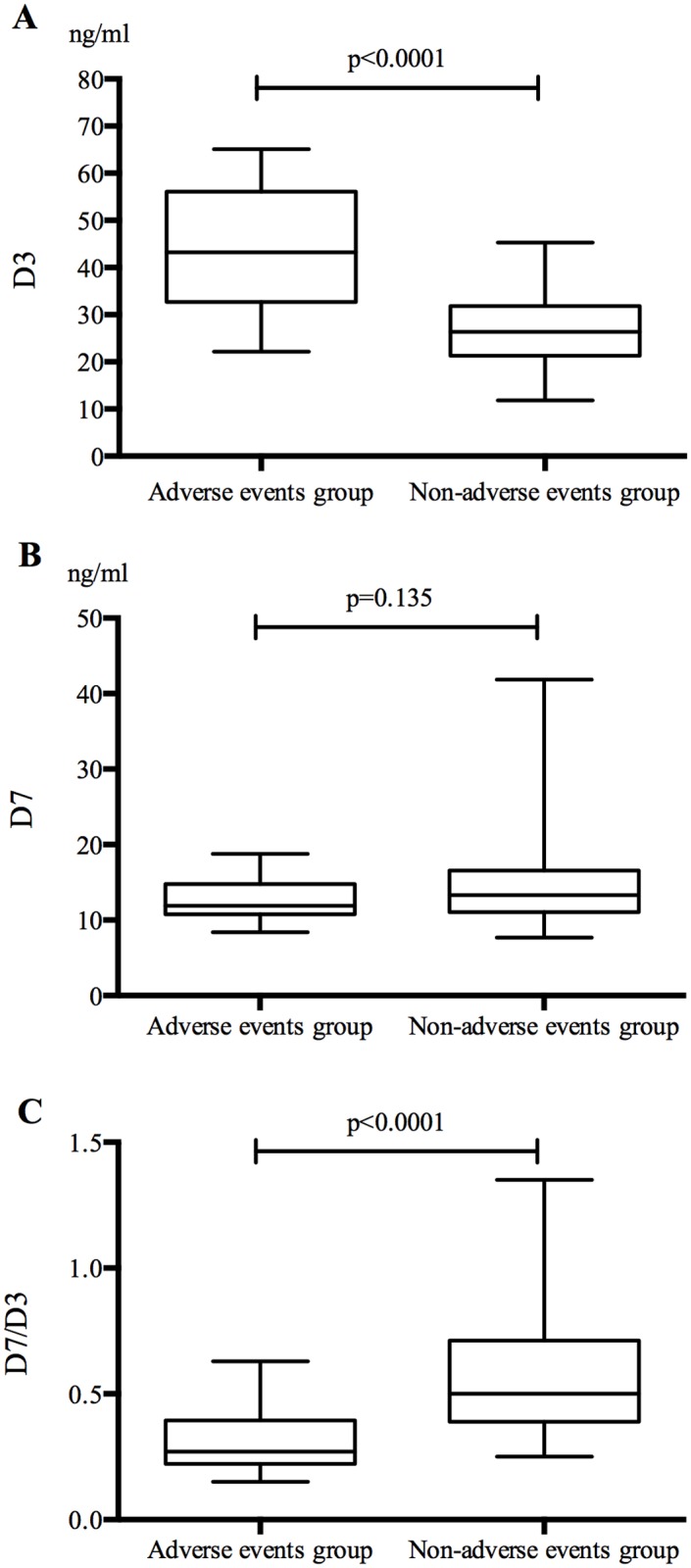
Comparison of the catestatin levels between adverse events group and non-adverse events group. Box plots showing the distribution of concentrations of plasma catestatin in two groups (n1 = 26, n2 = 74) on ER (A), D3 (B) and D7 (C). Boxes specify inter-quartile ranges; the horizontal bars inside the boxes indicate medians; the upper and lower whiskers get to the most distant value within the 95% of the distribution. The p-values for the comparison of the groups are also indicated.

Patients were stratified into two groups (the adverse events group and the non-adverse events group) according to whether patients recruited showed up cardiac adverse events during our study. Table 2 summarizes and compares patient characteristics from these two groups. During the follow-up of 65 months (54.98±19.20 months), 26 patients had adverse events. The levels of catestatin on ER and D3 were significantly higher in adverse events group (19.4±6.7ng/ml, 44.6±13.0ng/ml, respectively) than those in non-adverse events group (15.8±4.5ng/ml, p = 0.003, 26.1±7.5ng/ml, p<0.0001, respectively), but the ratio of D7/D3 was much lower in adverse events group (0.31±0.13) than that in non-adverse events group (0.59±0.25, p<0.0001) (Fig 2), because the p values of D3 and D7/D3 were lower than that of ER, it’s reasonable to hypothesize that the differences of them are more closed to the real, so we chose these two parameters to conduct the subsequent analysis. For other factors that might have influence on the outcome of AMI, the differences of body mass index, LDL cholesterol, percentage of diabetes mellitus and current smokers are significant (Table 2).

**Table 2:** Patient characteristics according to occurrence of adverse events.

Variables	Non-adverse events group (n = 74)	Adverse events group (n = 26)	p value
**Age (years)**	61.8±14.8	63.0±11.8	0.703
**Gender (M/F)**	62/12	22/4	1.000
**Body mass index (kg/m2)**	24.9±2.2	26.9±3.7	0.012
**HRs**	83±13	82±9	0.788
**Systolic blood pressure (mmHg)**	133.0±18.4	138.2±16.0	0.202
**Diastolic blood pressure (mmHg)**	75.7±13.5	81.0±11.3	0.079
**Creatinine (μmol/l)**	97.7±18.9	95.0±12.9	0.504
**Fasting glucose (mmol/l)**	6.6±2.4	6.4±2.1	0.713
**LDL cholesterol (mmol/l)**	3.01±0.82	3.43±0.92	0.029
**LVEDD (cm)**	49.4±4.3	49.0±6.2	0.700
**LVEF (%)**	53.3±10.2	50.5±9.8	0.244
**Lesion Region-LAD, n (%)**	24 (32.4)	4 (15.4)	0.096
**Lesion Region-LCX n (%)**	24 (32.4)	10 (38.5)	0.577
**Lesion Region-RCA n (%)**	26 (35.2)	12 (46.1)	0.319
**Diabetes mellitus, n (%)**	22 (29.7)	0 (0)	0.040
**Hypertension, n (%)**	42 (56.8)	20 (76.9)	0.068
**Hypercholesterolemia, n (%)**	44 (59.5)	12 (46.2)	0.240
**Current Smoking, n (%)**	46 (62.2)	10 (38.5)	0.036
**Beta-blocker treatment, n (%)**	72 (97.3)	26 (100)	0.974
**ACEI/ARB treatment, n (%)**	56 (75.7)	22 (84.6)	0.344
**Aspirin treatment, n (%)**	68 (91.9)	25 (96.2)	0.464
**Plavix treatment, n (%)**	65 (87.8)	23 (88.5)	0.933
**Catestatin-ER (ng/ml)**	15.8±4.5	19.4±6.7	0.003
**Catestatin-D3 (ng/ml)**	26.1±7.5	44.6±13.0	<0.0001
**Catestatin-D7 (ng/ml)**	14.4±5.8	12.6±2.6	0.135
**Catestatin-D7/D3**	0.59±0.25	0.31±0.13	<0.0001

Values represent mean ± SD or the percent of the patients.

LVEDD, left ventricular end diastolic diameter; LVEF, left ventricular ejection fraction; LAD, left anterior descending coronary artery; LCX, left circumflex coronary artery; RCA, right coronary artery

### Difference of outcomes according to the value of D3, D7 and D7/D3 in patients with AMI

**Fig 3:**
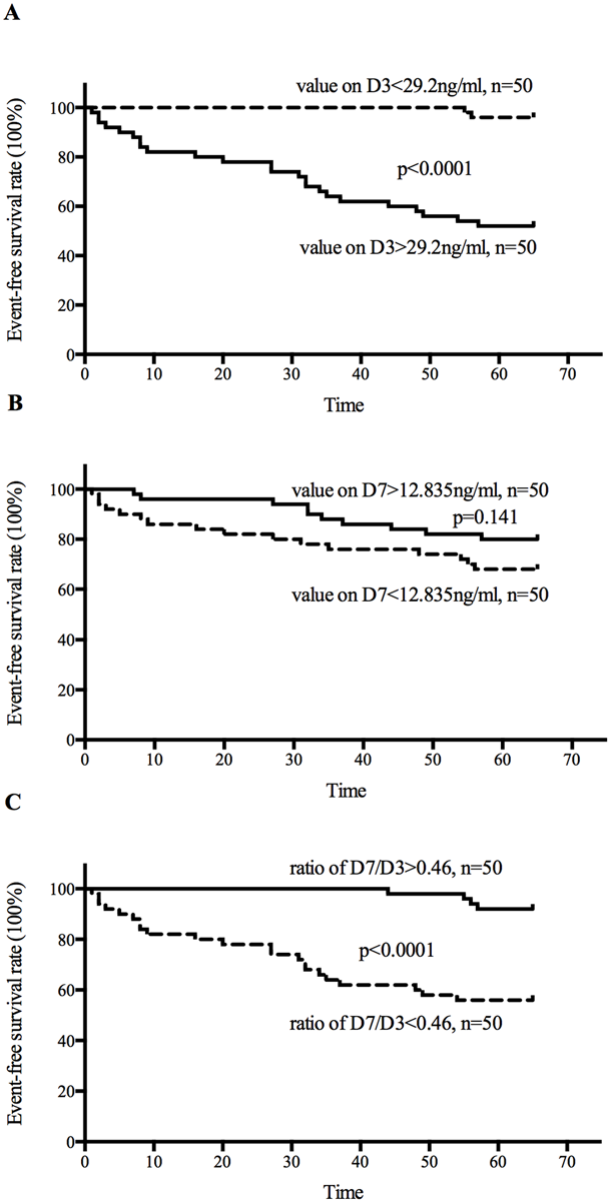
Kaplan-Meier survival curves. According to the median value of catestatin levels on D3 (A), D7 (B) and the ratio of D7/D3 (C), patients were separated into two groups respectively to analyze the event-free survival rate.

Patients were divided into 2 groups based on the median values of catestatin on D3 (29.2ng/ml), D7 (12.835ng/ml) and D7/D3 (0.46), event-free survival rates were measured using long-rank test, as shown with Kaplan-Meier survival curves, we found that the occurrence rate of adverse events was higher (p<0.0001) in the group in which the values of D3 were higher (>29.2ng/ml), but in the group in which the ratios of D7/D3 were lower (<0.46), but again, we didn’t see significant difference when analyzing values on D7 (Fig 3).

### Univariate and Multivariable Predictors of adverse events: Comparison of catestatin concentrations on D3, D7 and D7/D3 and other possible parameters

**Fig 4:**
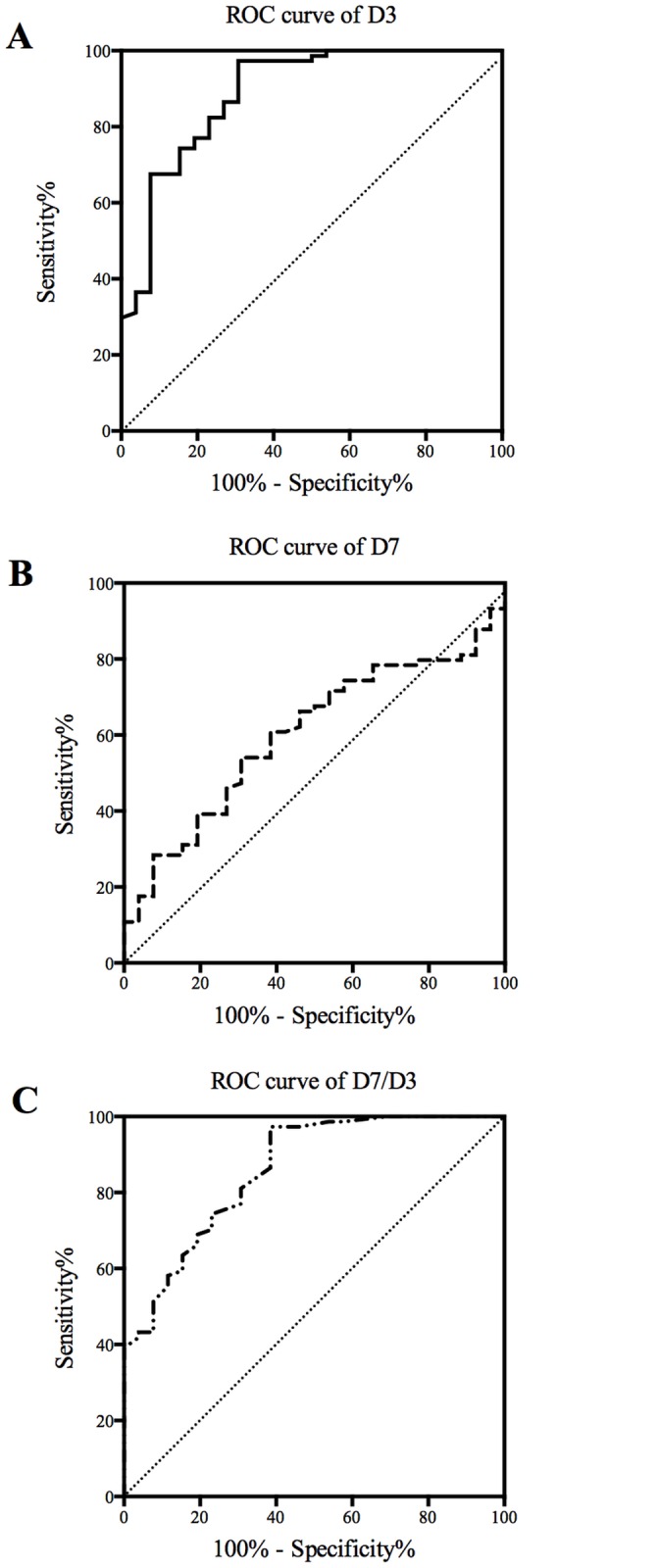
Receiver operating characteristic curves. The ability of catestatin values on D3 (A), D7 (B) and D7/D3 (C) to predict adverse events in patients with AMI was evaluated and their specificity as well as sensitivity were determined. The diagonal was shown in each diagram.

Eight clinical variables were analyzed using univariate and stepwise multivariable Cox proportional hazard regression analyses (Table 3). On stepwise multivariable analyses, only the value of logD3 was significantly independent predictor (p<0.0001). ROC curves of values on D3, D7 and D7/D3 demonstrating adverse events risks after AMI are shown in Fig 4. The area under curve of D3 is 0.888 (AUC = 0.888), the cut off level for D3 was determined as 32.95ng/ml, giving a sensitivity of 76.9% and specificity of 82.4%; the area under curve of D7 is 0.601 (AUC = 0.601), the cut off level for D7 was determined as 12.43ng/ml, giving a sensitivity of 61.54% and specificity of 60.81%; and the area under curve of D7/D3 is 0.860 (AUC = 0.860), the cut off level for D7/D3 was determined as 0.39, giving a sensitivity of 76.9% and specificity of 74.3%.

**Table 3:** Univariate and Multivariate Predictors of adverse events: comparison of catestatin concentrations on D3 and D7/D3 and other possible parameters.

Variables	Univariate		Multivariate	
	Chi-square	p value	Chi-square	p value
Age (years)	0.092	0.761		
Gender (male = 1)	0.051	0.882		
LVEDD (cm)	0.045	0.702		
LVEF (%)	1.460	0.227		
Creatinine (μmol/l)	0.486	0.486		
logD3	52.852	<0.0001	52.852	<0.0001
logD7	2.404	0.121		
log(D7/D3)	26.814	<0.0001		

## Discussion

Activation of the sympathetic nervous system, as assessed by circulating concentrations of catecholamines, is associated with reduced survival in patients with chronic heart failure [24]; greatly elevated plasma catecholamine levels in the acute and subacute phase after AMI also signify a poor prognosis [25], however, measurement of circulating catecholamines is rarely used for risk stratification, probably because of a cumbersome sampling procedure that involves insertion of an intravenous cannula and a period of rest in the supine position before blood collection [26]. Thus, a simple and reliable index that is less prone to rapid fluctuations in circulating levels, and that has similar or better prognostic value as catecholamines, would be useful.

The current study demonstrated the changes of plasma catestatin levels at certain time points during the first week in patients with AMI who underwent successful PCI within 12 hours from onset: catestatin levels decreased on admission, increased significantly on D3, but remained decreased on D7. In the 65 months' follow-up study, we found that circulating levels of catestatin in the first week after onset of AMI were associated with long-term adverse cardiac events. And it is also an independent prognostic indicator with high sensitivity and specificity. Since catestatin can be measured in serum or plasma collected with standard equipment, stored for prolonged period, and analyzed without tedious extraction procedures, developing the clinical use of catestatin might be of great importance.

Among many peptides derived from CHGA, catestatin was the only one responsible for inhibition of catecholamine release caused by nicotinic stimulation [9]. In recent years, CHGA has been found to be a sensitive marker of prognosis of morbidity and mortality in acute coronary syndromes (ACS), the mechanism for the predictive effects could probably contribute to the downstream product catestatin [27]. It is well established that catecholamine activation in patients with AMI, as measured by plasma adrenaline and noradrenaline, showed an early and rapid increase in the first 3 days and was restricted to the first 5–7 days [28], so the time points for blood sampling in our study were chosen according to the changes of catecholamines, which play an important role in a vicious circle that increases myocardial irritability and damage in AMI. In our study, it is speculated that the decreased catestatin levels observed at admission may be the response to the increased catecholamines levels, which might inhibit the release of catestatin; we also found the catestatin levels on D3 were the highest, which gave us the clue that the increased sympathoadrenal activities might increase catestatin release to promote negative feedback; moreover, when the catecholamines returned to normal levels within 5–7 days, the corresponding decrease of catestatin on D7 might further indicate the compensation mechanism of catecholaminergic activation, which might reduce the harmful effect of catecholamine. And dramatic change in patients with AMI indicates its possible involvement in the progress of AMI.

Initially identified as the most potent endogenous antagonist of nicotinic-cholinergic receptor, catestatin exerted antagonistic inhibition of nicotine-evoked catecholamines secretion in a non-competitive way [9], and it has been proved to be a multifunctional peptide with different mechanisms in the last decade, for example, its vasoactive and relevant anti-hypertensive properties have been extensively reviewed by a lot of researchers (e.g. [29]). In this study, the results indicated that the occurrence rate of cardiac adverse events in patients with AMI increased significantly as the catestatin levels elevated on D3, also, patients with higher catestatin levels seemed to have higher probability to develop adverse events. Here we surmised the increase of catestatin was the compensatory response of the sympathetic nerve activity, which release catecholamine and do harm to the myocardium, so the catestatin levels during the acute phase of AMI could reflect the degree of sympathetic nervous system activation, which gives its role to be a good prognostic factor of adverse events. Furthermore, there should be a balance between catecholamines and catestatin, but it might be insufficiently compensatory at a higher level (acute phase of AMI here), and this is still not enough to compensate the damage to the body caused by sympathetic and catecholamine toxicity.

Though most evidences showed catestatin is related with cardiovascular diseases, whether it is a protective factor and the underlying mechanisms are still not well elucidated. Previous research indicated that in isolated murine heart, catestatin (75nM) could significantly reduce the infarction size after ischemia and improve the post-ischemia left ventricular systolic and diastolic function [20]. But opposite to the effect mentioned above, some evidences showed both wild type (WT) and Pro370Leu (P370L) variant catestatin (100nM) increased infarction size, through inhibiting phosphorylation of Akt and its downstream targets, BAD and FoxO1, increasing the cell death [30]. The inconformity of those studies might be a result of different animal models, variants of catestatin and different concentration of catestatin. Since most damage happened during reperfusion after ischemia, this period is crucial for prognosis. In our study, we failed to reveal obvious relationship between catestatin and peak CK-MB or LVEF (S1 File), whether there is a correlation between catestatin and infarct size of heart remains to be elucidated in a relatively larger sample size, and more researches about the function of catestatin are needed to provide more clues about the role of catestatin in AMI and post-ischemia reperfusion.

There were still some notable limitations in this study. First, only 100 AMI patients had been recruited for the follow-up research, which is a relatively small group; second, this is also only one center study; third, there are some papers [31,32] which demonstrated the relationship between lipid mobilization and catestatin, but in our study, the mean value of BMI of participants was high and we also lost some of the BMI data, which made it confused to analyze; At last, a careful echocardiographic analysis at longer time might provide more useful information about the prognostic value of catestatin.

In conclusion, plasma catestatin levels changed dramatically during the first week in patients with AMI, suggesting an important role of catestatin in the progress of AMI. In addition, patients who had adverse events after AMI showed higher catestatin levels on D3, and when separating into 2 groups according to the median value on D3, the group with higher levels had more adverse events, at last, the multivariable Cox proportional hazard analyse also suggested that the level on D3 was an independent predictor, all of which determined the predictive role of catestatin level on D3 in AMI, but the particular underlying mechanism should also be studied further.

## Supporting Information

S1 File Correlations between catestatin-D3 levels and peak CK-MB as well as LVEF. The correlation between catestatin levels on D3 and peak CK-MB as well as LVEF were determined using Spearman or Pearson correlation analyses, the p values were 0.999, 0.128, respectively.
(TIFF)

S2 File Patients Data. (XLS)

S3 File Control Subjects Data. (XLS)
