# Supplementary figures and images for: Correlation of Plasma Catestatin Level and the Prognosis of Patients with Acute Myocardial Infarction
Source: PLoS One. 2015 Apr 7;10(4):e0122993. doi: 10.1371/journal.pone.0122993 (PMC4388679; doi:10.1371/journal.pone.0122993)

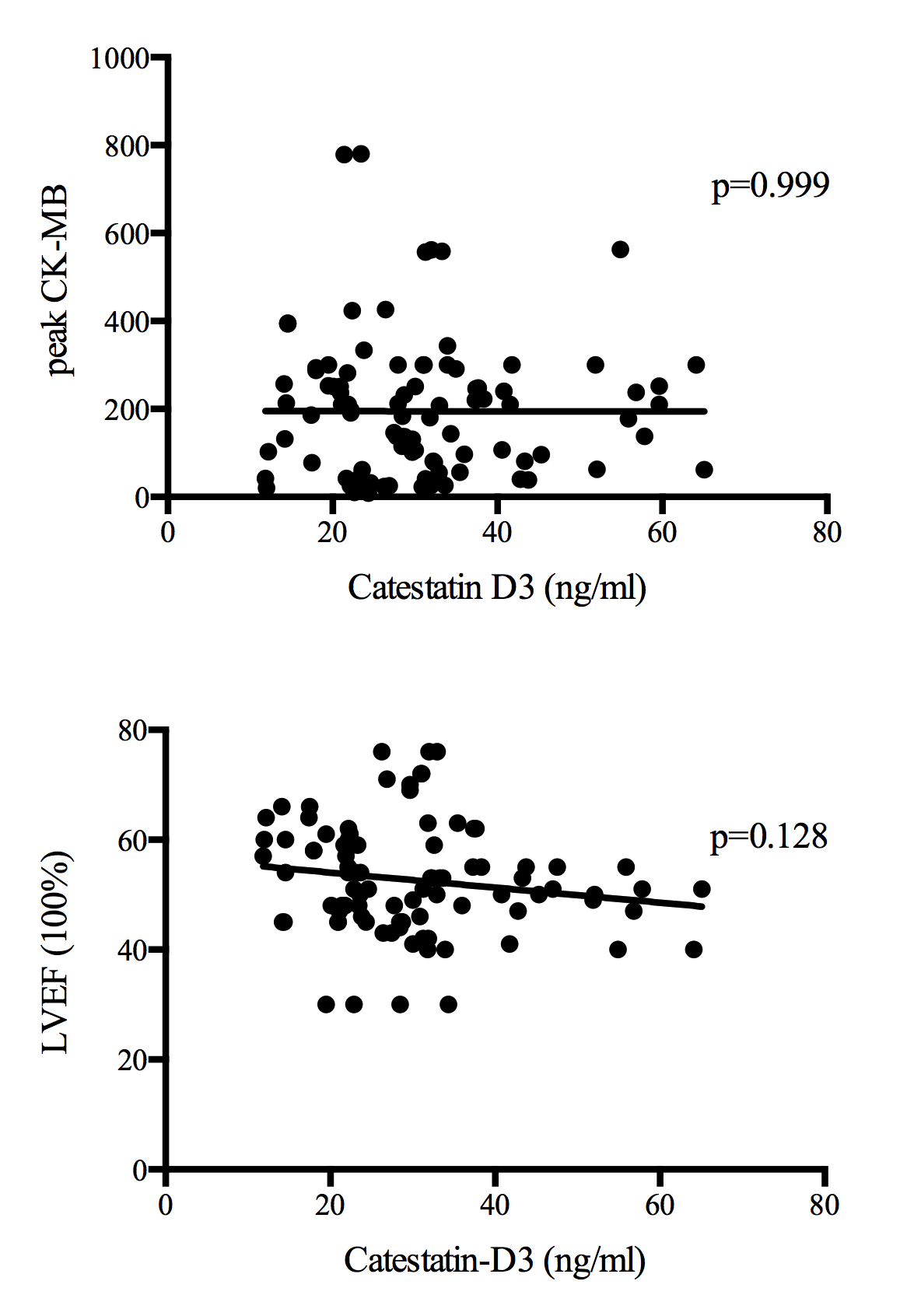

Supplement: S1 File — The correlation between catestatin levels on D3 and peak CK-MB as well as LVEF were determined using Spearman or Pearson correlation analyses, the p values were 0.999, 0.128, respectively. (TIFF) [file pone.0122993.s001.tiff]
